# Alleviation of diabetic nephropathy by zinc oxide nanoparticles in streptozotocin‐induced type 1 diabetes in rats

**DOI:** 10.1049/nbt2.12026

**Published:** 2021-03-10

**Authors:** Ghada Alomari, Bahaa Al‐Trad, Salehhuddin Hamdan, Alaa A. A. Aljabali, Mazhar Salim Al Zoubi, Khalid Al‐Batanyeh, Janti Qar, Gregory J. Eaton, Almuthanna K. Alkaraki, Walhan Alshaer, Saja Haifawi, Khairunadwa Jemon, Dinesh Kumar Chellappan, Kamal Dua, Murtaza M. Tambuwala

**Affiliations:** ^1^ Department of Bioscience, Faculty of Science Universiti Teknologi Malaysia Johor Bahru Johor Malaysia; ^2^ Department of Biological Sciences Yarmouk University Irbid Jordan; ^3^ Department of Pharmaceutics and Pharmaceutical Technology Faculty of Pharmacy Yarmouk University Irbid Jordan; ^4^ Department of Basic Medical Sciences Faculty of Medicine Yarmouk University Irbid Jordan; ^5^ Department of Biological Sciences College of Science and Mathematics Rowan University Glassboro New Jersey USA; ^6^ University of Jordan Cell Therapy Center Amman Jordan; ^7^ Department of Life Sciences School of Pharmacy International Medical University Kuala Lumpur Malaysia; ^8^ School of Biomedical Sciences and Pharmacy The University of Newcastle Callaghan Australia; ^9^ School of Pharmacy and Pharmaceutical Science Ulster University Coleraine UK

## Abstract

This study examines the effect of nanoparticles with zinc oxides (ZnONPs) on diabetic nephropathy, which is the primary cause of mortality for diabetic patients with end‐stage renal disease. Diabetes in adult male rats was induced via intraperitoneal injection of streptozotocin. ZnONPs were intraperitoneally administered to diabetic rats daily for 7 weeks. Diabetes was associated with increases in blood glucose level, 24‐h urinary albumin excretion rate, glomerular basement membrane thickness, renal oxidative stress markers, and renal mRNA or protein expression of transforming growth factor‐*β*1, fibronectin, collagen‐IV, tumour necrosis factor‐*α* and vascular endothelial growth factor‐A. Moreover, the expression of nephrin and podocin, and the mRNA expression of matrix metalloproteinase‐9 were decreased in the diabetic group. These changes were not detected in the control group and were significantly prevented by ZnONP treatment. These results provide evidence that ZnONPs ameliorate the renal damage induced in a diabetic rat model of nephropathy through improving renal functionality; inhibiting renal fibrosis, oxidative stress, inflammation and abnormal angiogenesis; and delaying the development of podocyte injury. The present findings may help design the clinical application of ZnONPs for protection against the development of diabetic nephropathy.

## 1 INTRODUCTION

Diabetic nephropathy (DN) is a significant diabetic microvascular complication. The incidence of DN has continued to increase with the expanding cohort of diabetes patients around the world [1] and has become a leading cause of chronic kidney disease in developed countries [2]. Usual DN histological changes include thickening in both the tubular basement membrane (TBM) and the glomerular basement membrane (GBM), mesangial expansion, and afferent and efferent arteriolar hyaline deposition [3]. Mesangial enlargement, GBM thickening, and TBM thickening are consequences of increased removal and/or decreased degradation of extracellular matrix (ECM) proteins such as collagens, laminin, and fibronectin [3, 4]. Moreover, it has been established that podocyte apoptosis, necrosis or the loss of their adhesive interaction lead to podocyte detachment from GBM. These cellular pathologies underlie the progression of DN and cause leakage of albumin into the urine [3, 4].

Under chronic hyperglycemia, many molecular pathways may affect DN progression. These pathways include ECM protein deposition, cellular hypertrophy, oxidative stress, inflammation and apoptosis. Moreover, chronic hyperglycemia enhances flux into the polyol, and hexosamine pathways increase the formation of advanced glycation end products and upregulate activation of protein kinase C and transforming growth factor *β* (TGF*β*) signalling pathways [5]. Abnormal angiogenesis also plays a central role in DN progression [4, 6].

The essential trace element zinc (Zn) has different roles in many biological processes, including as a catalytic cofactor, in immune response, in the second messenger system, and in the structural functions of numerous proteins [7, 8]. Systemic Zn deficiency has been associated with a high incidence of diabetes and advancing DN [9, 10, 11]. In addition, Zn deficiency can cause albuminuria enhancement and ECM protein expression through the activation of renal interstitial fibroblasts via the TGF‐*β*/Smad2/3 pathway, which is correlated with diabetic renal interstitial fibrosis [12]. Therefore, Zn supplementation has been shown to have potent anti‐diabetic activity [10], has alleviated the progression of DN [13], and seems to ameliorate glycaemic control in type 1 and type 2 diabetes through its insulin‐like effects and by protecting pancreatic *β* cells from being destroyed by immune cells and cytokines [7].

Recently, nanoparticles have been widely used in biomedical applications and have demonstrated several advantages as therapeutic materials because they can pass through biological barriers and enhance the bioavailability of therapeutic agents [14, 15]. Recent studies showed that zinc oxide nanoparticles (ZnONPs) had anti‐hyperglycemic, anti‐oxidative stress, and anti‐inflammatory effects in a diabetic animal model [16, 17, 18]. This study evaluates the possible therapeutic effect of ZnONPs on DN in a streptozotocin (STZ)‐induced rat model of type 1 diabetes.

## 2 MATERIALS AND METHODS

### 2.1 Experimental animals and protocols

The Animal Ethics Committee approved all animal procedures at Yarmouk University (Irbid, Jordan). Adult male Wistar rats (weighing 250 g ± 20 g) were kept under specific pathogen‐free conditions in the Yarmouk University animal housing facility. After one week of adaptation followed by overnight fasting, rats were intraperitoneally injected with 55 mg/kg STZ (Chem Cruz, U‐9889) to induce type I diabetes. Rats received a drinking water supplemented with sucrose for control of early death secondary to hypoglycaemic shock (15 g/L) for 48 h. Three days after STZ injection, rats with a blood glucose level above 250 mg/dl were considered diabetic [19]. Three different groups were established randomly: non‐diabetic group (ND, *n* = 6), diabetic group without treatment (*D*, *n* = 6) and diabetic group with daily intraperitoneal injections of 2.5 mg/kg ZnONPs (*D* + ZnONPs, *n* = 6) for 7 weeks. ZnONPs size <100 nm (TEM) were purchased from Sigma‐Aldrich (Germany, Catalogue No. 721077). The intraperitoneal route of administration of ZnONPs in the present study was selected based on a previous study that reported that intraperitoneal injected ZnONPs were more effectively distributed in the kidneys than was their distribution following oral administration [20]. The dose used in this study was selected depending on different studies that reported non‐toxic, protective, or recovering roles of ZnONPs on different organs and diseases [21, 22, 23, 24, 25]. In the current study, daily administration of 2.5 mg/kg ZnONPs in animals was well tolerated for 7 weeks (no significant changes in intake of feed, behaviour or mortality rates). Furthermore, there were no significant effects of hepatic function analysis from 2.5 mg/kg ZnONPs treatment on serum alanine aminotransferase and aspartate aminotransferase levels (Supplement 1).

### 2.2 Renal function and biochemical analysis

After 7 weeks of ZnONPs treatment, rat urine collection was performed using individual metabolic cages for 24 h. Urinary albumin concentration was measured using the Albumin Rat ELISA kit (cat no. ab235642, Abcam, UK) according to manufacturer instructions. Using commercially available kits, blood glucose and blood urea nitrogen levels (BUN) were measured (ARCOMEX, Jordan). The rat kidney weight (mg) to body weight (g) ratio was calculated to obtain the kidney weight index (KWI).

### 2.3 Assessment of renal oxidative stress markers

Superoxide dismutase (SOD) (Cayman Chemical, USA), glutathione peroxidase (GPx) (Cayman Chemical, USA) and catalase (CAT) (Abbexa Ltd, UK) enzyme activity were measured in homogenised kidney tissue according to each kit manufacturer's procedures and recommendations. Renal tissue malondialdehyde (MDA) level, a lipid peroxidation marker, was assessed by a method described by Buege and Aust [26].

### 2.4 Renal morphology and immunohistochemistry

Paraffin sections were prepared and stained with H&E and periodic acid Schiff (PAS; Sigma‐Aldrich, USA). Prepared slides were assessed for any histopathological change under a light microscope. For ultrastructural evaluation, renal cortical tissues were fixed in 2.5% glutaraldehyde and postfixed in sodium cacodylate‐buffered 1% OsO_4_ (v/v %), dehydrated and embedded in spur's resin. Ultrathin sections were processed on an ultratome (Reichert‐Jung Ultratome) and contrasted using uranyl acetate and lead citrate. Sections were examined under a Hitachi electron microscope (HT7700‐Tokyo). The GBM thickness was measured and calculated as previously detailed [27]. Podocyte number, and foot process width (FPW) was calculated according to the method of a previous study [28].

Immunohistochemical staining was performed in 4 µm paraffin‐embedded sections with specialised detection Kit (Mouse and Rabbit Specific HRP/DAB IHC–Micropolymer, Abcam) according to the manufacturer protocol using primary antibodies against ECM protein collagen IV (1:100; Abcam/UK; Cat. No. ab6586), the specific podocyte markers nephrin and podocin (1:100; Abcam/UK; Cat. No. ab183099 and 1:50; Abcam/UK, Cat. No. ab50339, resp.), the fibrotic marker TGF‐*β*1 (1:100; Santa Cruz/USA; Cat. No. sc‐146) and vascular endothelial growth factor‐A (VEGF‐A; 1:100; Santa Cruz/USA; Cat. No. sc‐7269). Negative controls were run by replacing the primary antibody with PBS. Under an OPTIKA microscope, section scoring and analyses were performed with an observer masked to treatment groups. The mean optical density was quantified by the use of Fiji ImageJ software in a positively stained field.

### 2.5 Renal morphology and immunohistochemistry

A cDNA reverse transcription kit (Takara, China) was used to prepare the cDNA template. Quantitative real‐time RT‐PCR was conducted using the Line‐Gene 9600 Real‐Time PCR system (Bioer Technology Co, Binjiang, China) with a SYBR green PCR master mix (Takara, China). PCR primers (Table 1) for TGF‐*β*1, VEGF‐A, MMP‐9, fibronectin, and TNF‐*α* were purchased from Integrative DNA Technologies (Coralville, USA). Amplification conditions were 95°C for 2 min and 45 cycles at 95°C for 30 s and 60°C for 30 s. The mRNA levels were standardised as a non‐regulated GAPDH mRNA reference gene. Fold changes in mRNA expression were determined using the 2−ΔΔCT method [29].

**TABLE 1:** Sequences of primers used for quantitative real‐time RT‐PCR

Primer	Sequence
GAPDH	Left: 5′‐ATG GTG AAG GTC GGT GTG‐3′
	Right: 5′‐GAA CTT GCC GTG GGT AGA‐3′
VEGF	Left: 5′‐CGA ACA GAG AGA GGG ACA GG ‐3′
	Right: 5′‐GTC TGT CTG TCT GTC CGT CA ‐3′
TGF‐*β*	Left: 5′‐CTT CCC CTC CGA AAC TGT CT ‐3′
	Right: 5′‐AGG GTC TGT AGA AAG TGG GC‐3′
Collagen IV	Left: 5′‐ TTG GCT TTC CTG GTA GTC GT ‐3′
	Right: 5′‐ CAA CCT TTC CTG CTT CAC CC ‐3′
Fibronectin	Left: 5′‐ TGA CCG ACG CTA CAG AAA CT ‐3′
	Right: 5′‐ TTG AGC GTG TAC AGG TGG AT ‐3′
TNF‐*α*	Left: 5′‐ TTC GGA ACT CAC TGG ATC CC ‐3′
	Right: 5′‐ GGA ACA GTC TGG GAA GCT CT ‐3′
MMP‐9	Left: 5′‐AGG ATG GTC TAC TGG CAC AC ‐3′
	Right: 5′‐GTG CAG GAC AAA TAG GAG CG ‐3′

Abbreviations: MMP‐9, matrix metalloproteinase‐9; TNF‐*α*, transforming growth factor *α*; TNF‐*β*, transforming growth factor *β*; VEGF, vascular endothelial growth factor.

### 2.6 Statistical analysis

Statistical analysis was performed using GraphPad Prism version 7.00 (GraphPad, San Diego, CA). All results were expressed as means ± SEM. In the one‐way ANOVA, the significance of different groups was determined. Significant differences were tested using an LSD post hoc test for multiple comparisons (*p* values < 0.05).

## 3 RESULTS

### 3.1 Zinc oxide nanoparticle treatment resulted in decreased blood glucose level and improved renal function in diabetic nephropathy

**FIGURE 1:**
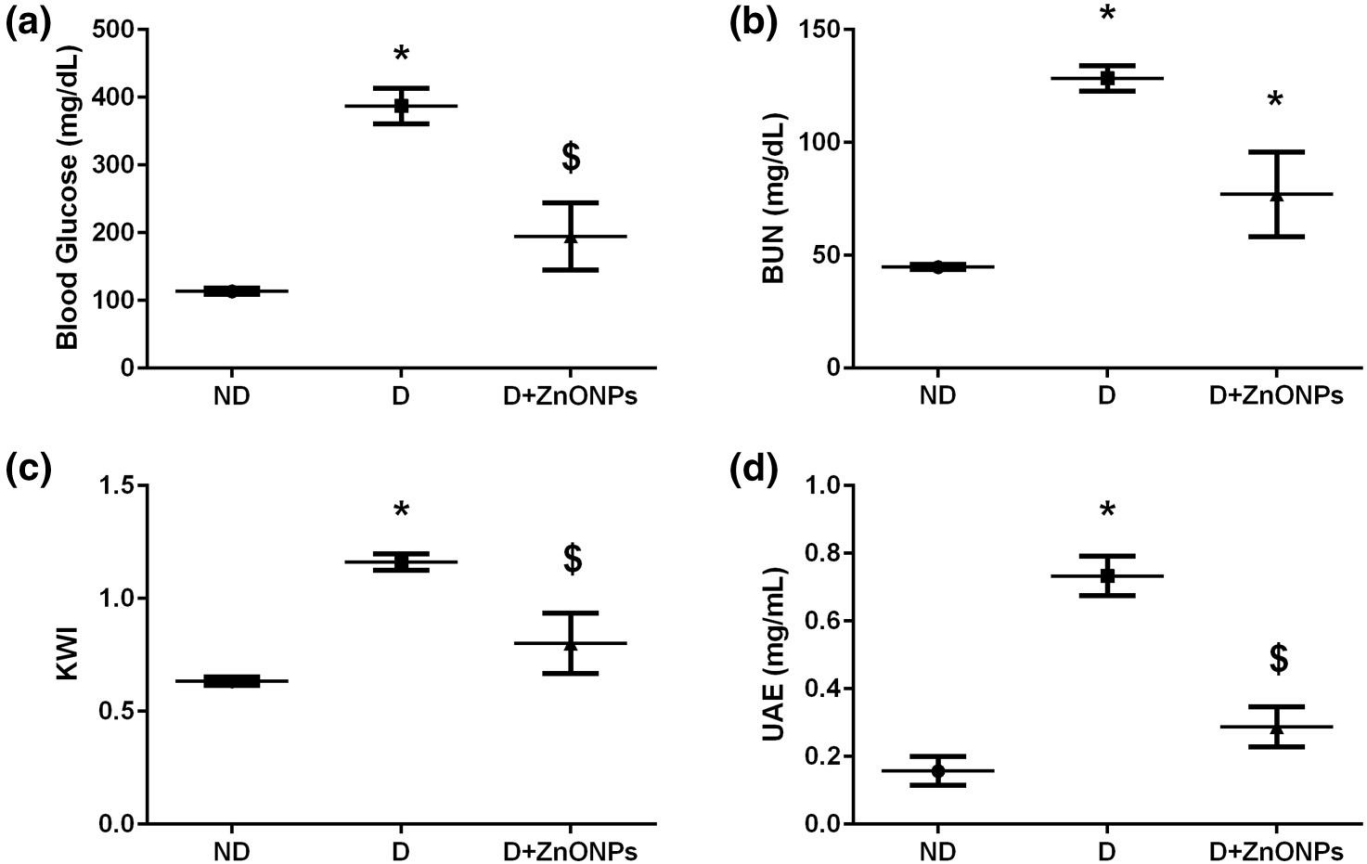
Renal function and biochemical analysis: blood glucose level (a), BUN (b), KWI (c), UAE (d). All values are expressed as mean ± SEM. Symbol (*) denotes significant differences (*P* < 0.05) with non‐diabetic, symbol ($) denotes significant differences (*P* < 0.05) with diabetic group. Abbreviations: ND, non‐diabetic; D, diabetic; D + ZnONPs, zinc oxide nanoparticle–treated diabetic group; BUN, blood urea nitrogen, KWI, kidney weight/body weight index; UAE, urine albumin excretion rate

After 7 weeks of STZ administration, diabetic rats showed significant increases in blood glucose, BUN level, and KWI compared with the non‐diabetic (ND) group (Figure 1). Albuminuria is considered a hallmark for the progression of renal disease, and the increase in the urine urinary albumin excretion rate (UAE) in addition to the BUN level and KWI are all indicators for the development of DN in diabetic rats [30]. ZnONP treatment significantly decreased blood glucose level and improved renal function in the *D* + ZnONPs group (*P* < 0.05; Figure 1 a–d).

### 3.2 Zinc oxide nanoparticle treatment attenuated renal histopathology injury

**FIGURE 2:**
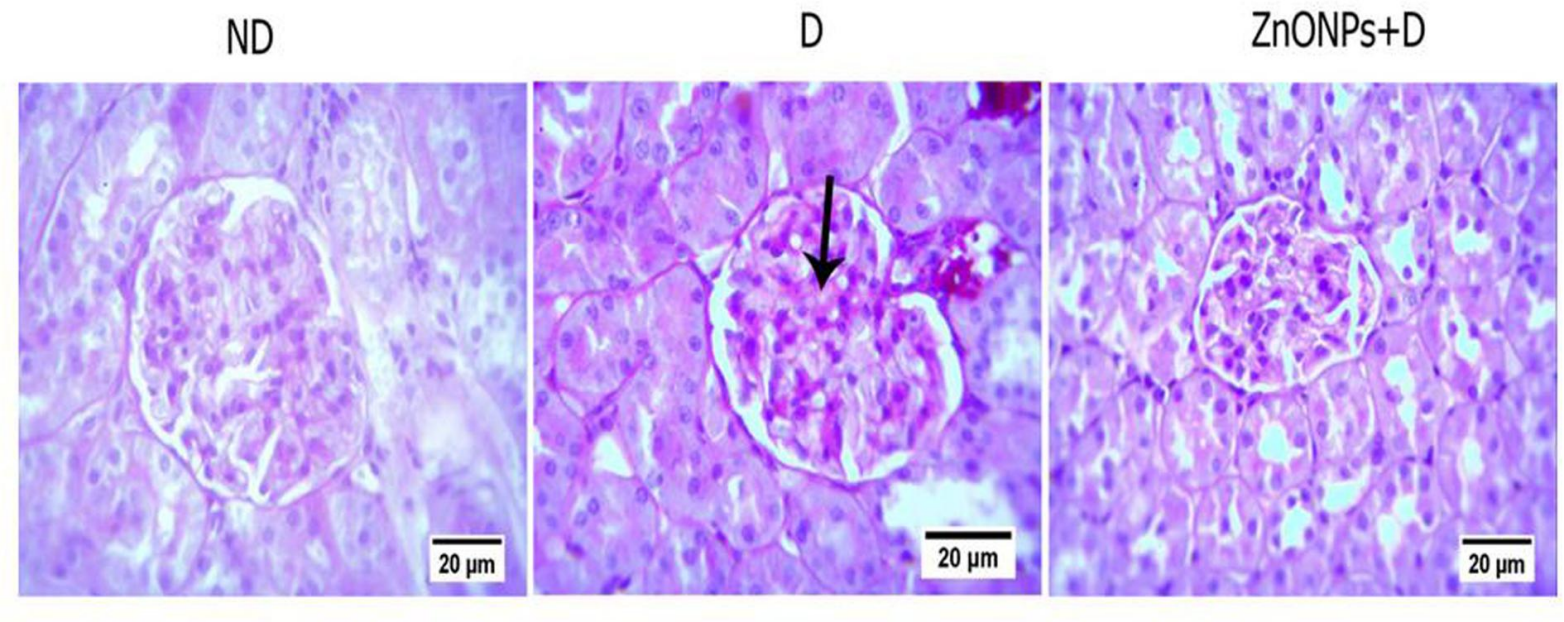
Representative PAS‐stained cortical renal tissue (magnification ×400). Sections from diabetic rats show mesangial expansion and basement membrane thickening comparing with the ND group, ZnONP treatment attenuated these renal damages. Arrow: mesangial expansion. ND, non‐diabetic; D, diabetic; ZnONPs + D, zinc oxide nanoparticle–treated diabetic group

**FIGURE 3:**
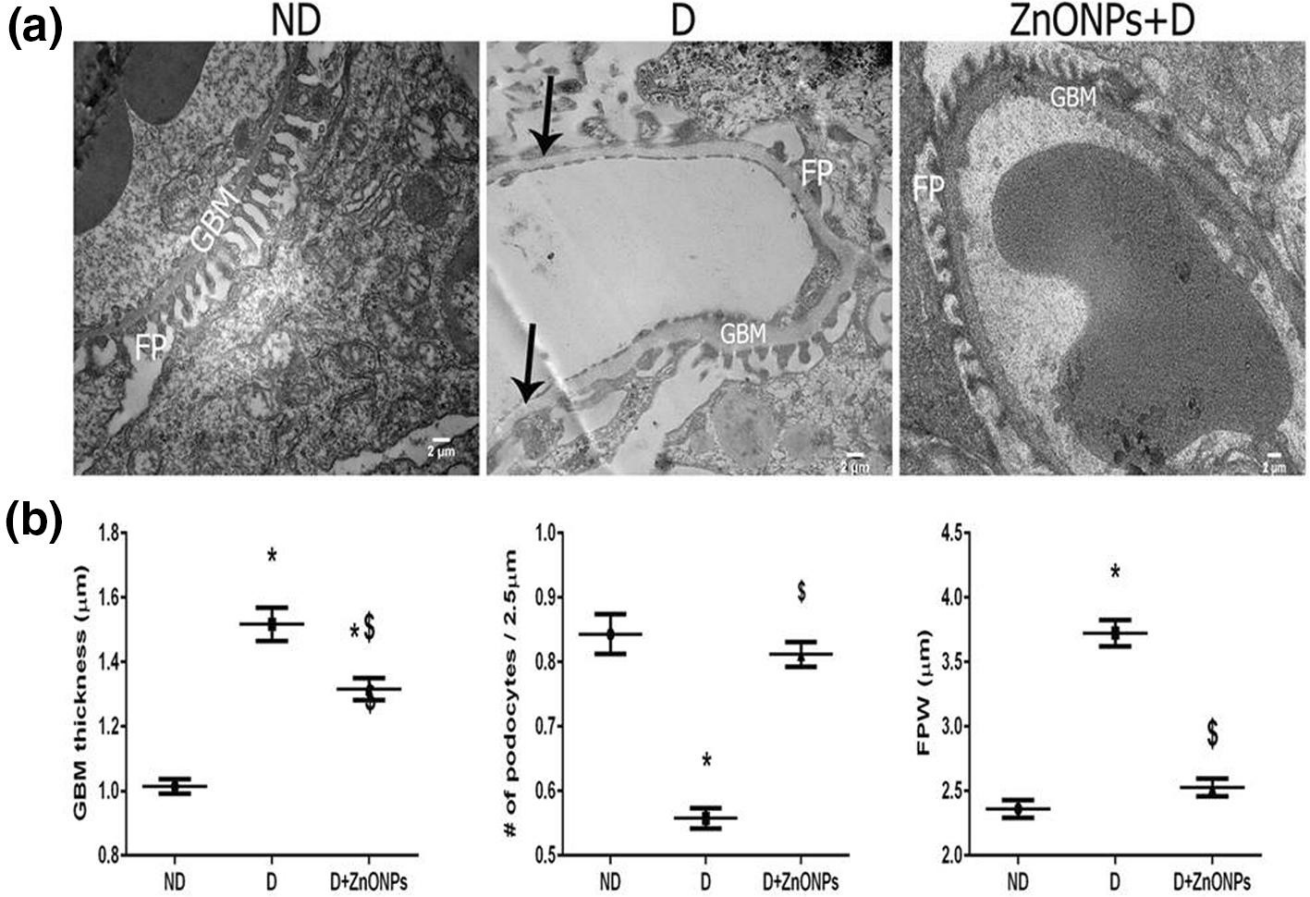
(a) Representative electron microscopy images of podocyte foot processes and the GBM. White arrow: foot process effacement (magnification ×6000); (b) glomeruli were evaluated to quantify the GBM thickness, number of podocyte and FPW. Data are expressed as means ± SEM (*n* = 6/each group). Symbol (*) denote significant differences (*P* < 0.05) with non‐diabetic, symbol ($) denotes significant differences (*P* < 0.05) with diabetic group. Abbreviations: GBM, glomerular basement membrane; FP, foot process; ND: non‐diabetic; D, diabetic; ZnONPs + D, zinc oxide nanoparticle–treated diabetic group

As shown in the PAS stain in Figure 2, the diabetic rats showed GBM thickening and mesangial matrix expansion compared with ND rats. Electron micrograph studies determined structural changes in podocyte morphology and GBM thickening. The ZnONP‐treated diabetic rats exhibited a significant improvement in ultrastructural abnormalities of the kidney, such as GBM thickening, loss of podocytes, and podocyte foot process effacement compared with the D group (*P* < 0.05; Figure 3a and b.

### 3.3 Zinc oxide nanoparticle treatment restores the expression of podocyte markers (nephrin and podocin)

**FIGURE 4:**
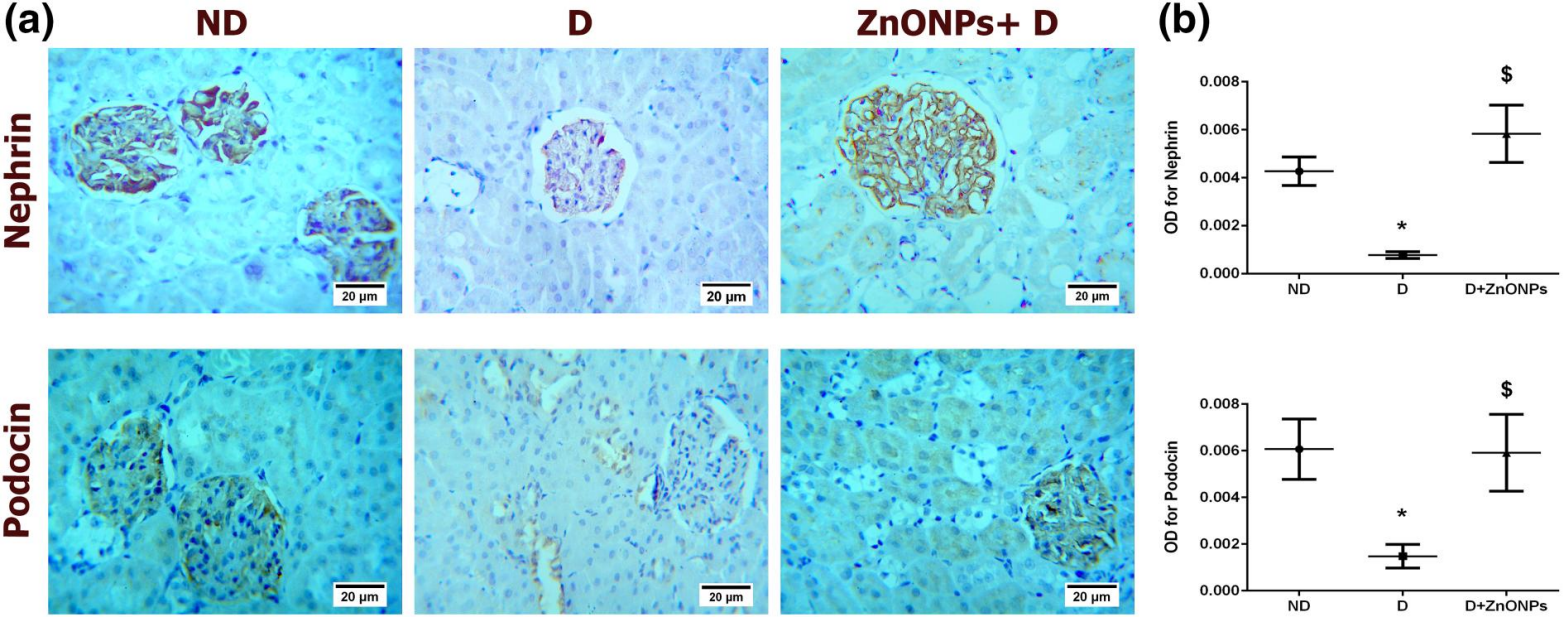
(a) Representative nephrin and podocin immunohistochemical staining of the kidney sections (haematoxylin staining; magnification ×400). (b) Quantification of nephrin and podocin immunohistochemistry results expressed as integral optical density of brown staining in the different groups. Data represent mean ± SEM. Symbol (*) denotes significant differences (*P* < 0.05) with non‐diabetic, symbol ($) denotes significant differences (*P* < 0.05) with diabetic group. Abbreviations: ND, non‐diabetic; D, diabetic; ZnONPs + D, zinc oxide nanoparticle–treated diabetic group; OD, optical density

The expression intensity of nephrin and podocin in the glomeruli were assessed by immunohistochemical staining (Figure 4a). In the D group, the expression intensity of nephrin and podocin were significantly decreased compared with the ND group (Figure 4b, *P* < 0.05). However, ZnONPs restored the immunohistochemical staining intensity of both markers in the ZnONPs + D group.

### 3.4 Zinc oxide nanoparticle treatment reduced renal expression of fibrosis markers in diabetic nephropathy

**FIGURE 5:**
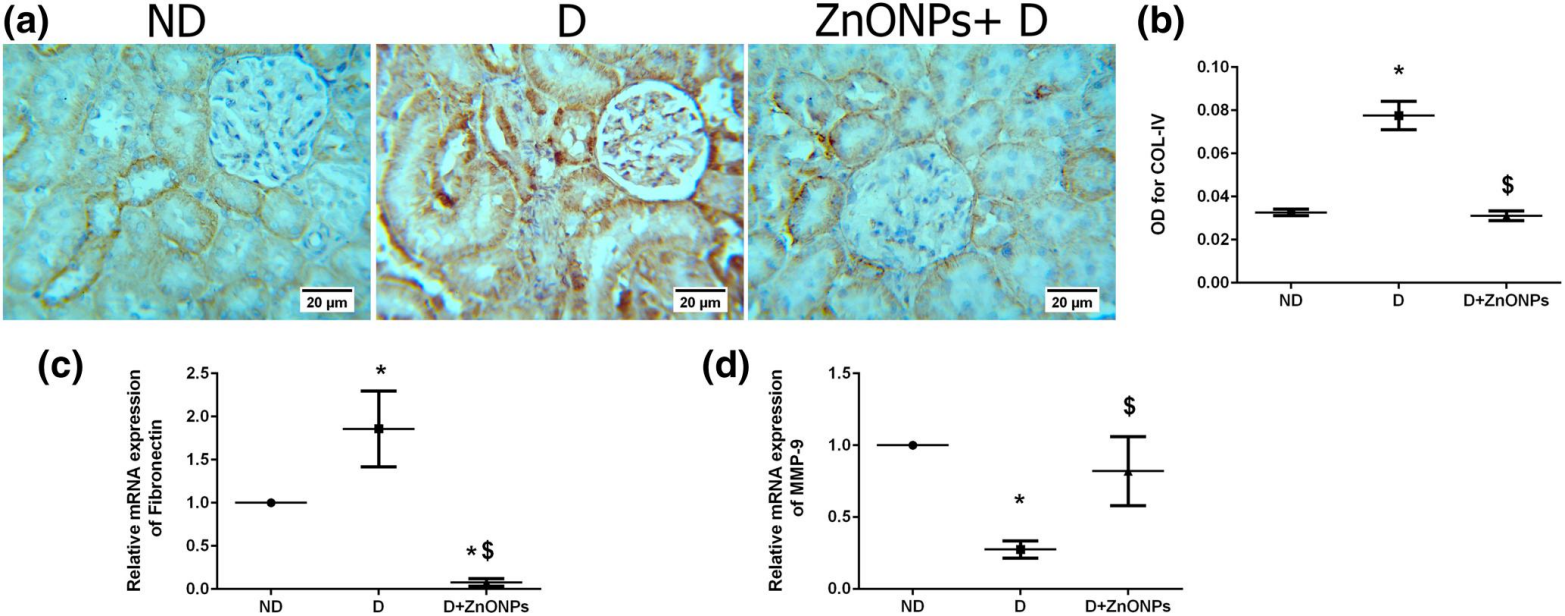
(a) Representative COL‐IV immunohistochemical stain of kidney sections (haematoxylin staining; magnification ×400). (b) Quantification of COL‐IV immunohistochemistry results expressed as integral optical density of brown staining in the different groups. (c) mRNA fibronectin expression level. (d) mRNA MMP‐9 expression level data represent mean ± SEM. Symbol (*) denotes significant differences (*P* < 0.05) with non‐diabetic, symbol ($) denotes significant differences (*P* < 0.05) with diabetic group. Abbreviations: ND, non‐diabetic; D, diabetic; ZnONPs + D, zinc oxide nanoparticle–treated diabetic group; OD, optical density; COL‐IV, collagen IV; MMP‐9, matrix metalloproteinase‐9

To determine the effect of ZnONPs on ECM protein deposition in glomeruli, we analysed the expression of renal collagen IV and fibronectin, which are the major ECM proteins that lead to the development of mesangial matrix expansion in DN [31, 32]. Compared with the D group, ZnONP treatment significantly decreased glomerular collagen IV staining intensity (Figure 5a–b, *P* < 0.05) and fibronectin mRNA expression level in the ZnONPs + D group (Figure 5c, *P* < 0.05). In addition to increased deposition of ECM, glucose‐induced inhibition of ECM degrading enzymes such as MMP‐9 has been shown to contribute to the accumulation of mesangial ECM proteins in the diabetic kidney [33]. Our results showed that MMP‐9 mRNA expression was decreased in the D group compared with the ND group (Figure 5d, *P* < 0.05), and ZnONP treatment significantly rescued MMP‐9 mRNA levels in the ZnONPs + D group compared with that in the D group (*P* < 0.05).

**FIGURE 6:**
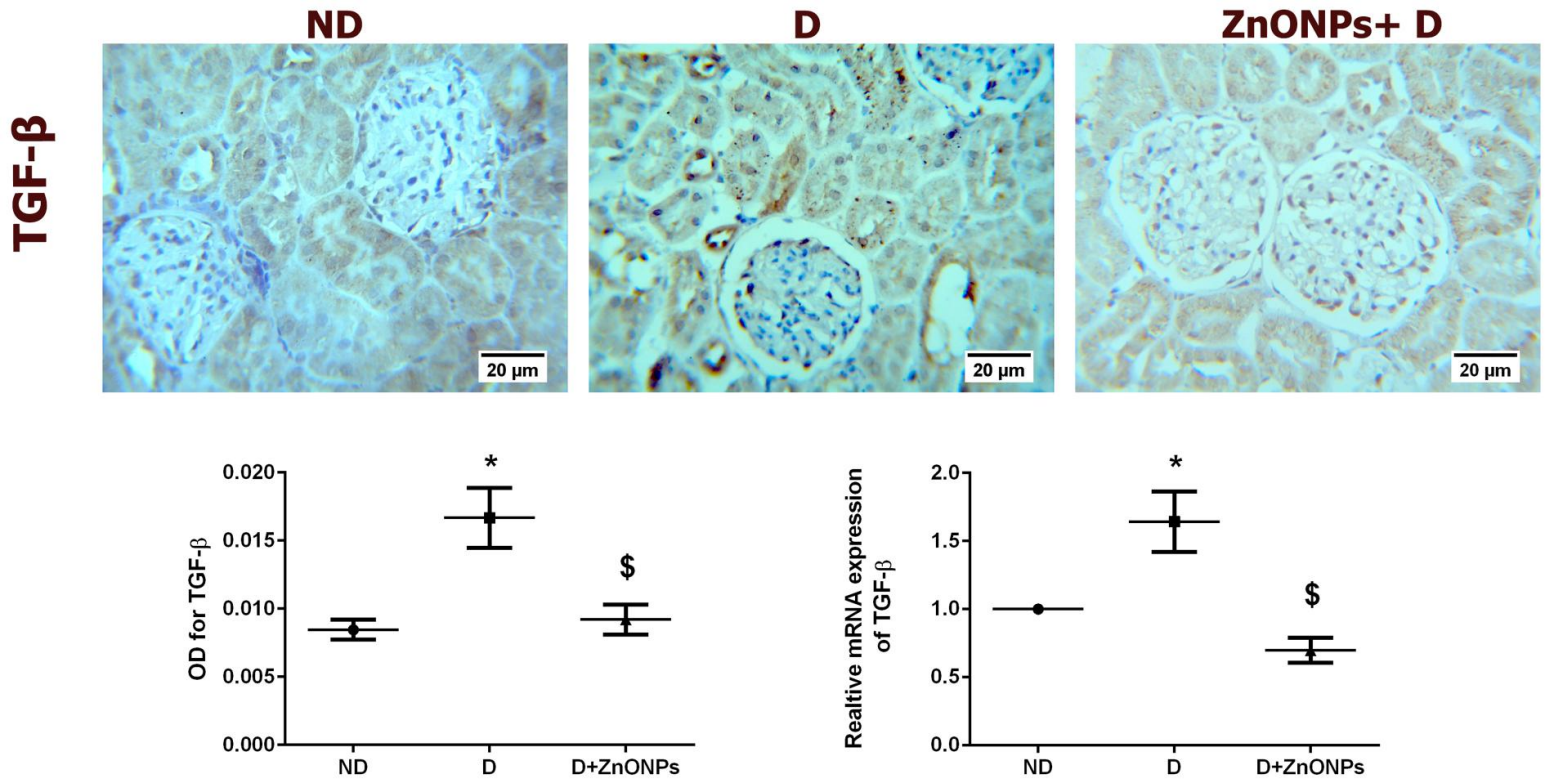
(a) Representative TGF‐*β*1 immunohistochemical staining of the kidney sections (haematoxylin staining; magnification ×400). (b) Quantification of the immunohistochemistry results expressed as integral optical density of brown staining in the different groups. (c) mRNA TGF‐*β*1 expression level. Data represent the mean ± SEM. Symbol (*) denotes significant differences (*P* < 0.05) with non‐diabetic, symbol ($) denotes significant differences (*P* < 0.05) with diabetic group. Abbreviations: ND, non‐diabetic; D, diabetic; ZnONPs + D, zinc oxide nanoparticle–treated diabetic group; OD, optical density; TGF‐*β*1, transforming growth factor *β*1

TGF‐*β*1 promotes renal cell hypertrophy and stimulates ECM accumulation, the two hallmarks of DN [34]. As presented in Figure 6a,b, immunohistochemistry analysis demonstrates that diabetic rats correlated with an overall increase in the intensity of immunostaining for TGF‐*β*1. Consistent with the immunohistochemical findings, the level of TGF‐*β*1 mRNA was significantly higher in the D group than in the ND group (Figure 6c; *P* < 0.05). However, treatment with ZnONPs resulted in a marked reduction in TGF‐*β*1 mRNA and protein expression levels in ZnONPs + D group compared with the D group.

### 3.5 Effect of zinc oxide nanoparticles on the renal expression of angiogenesis and inflammatory markers

**FIGURE 7:**
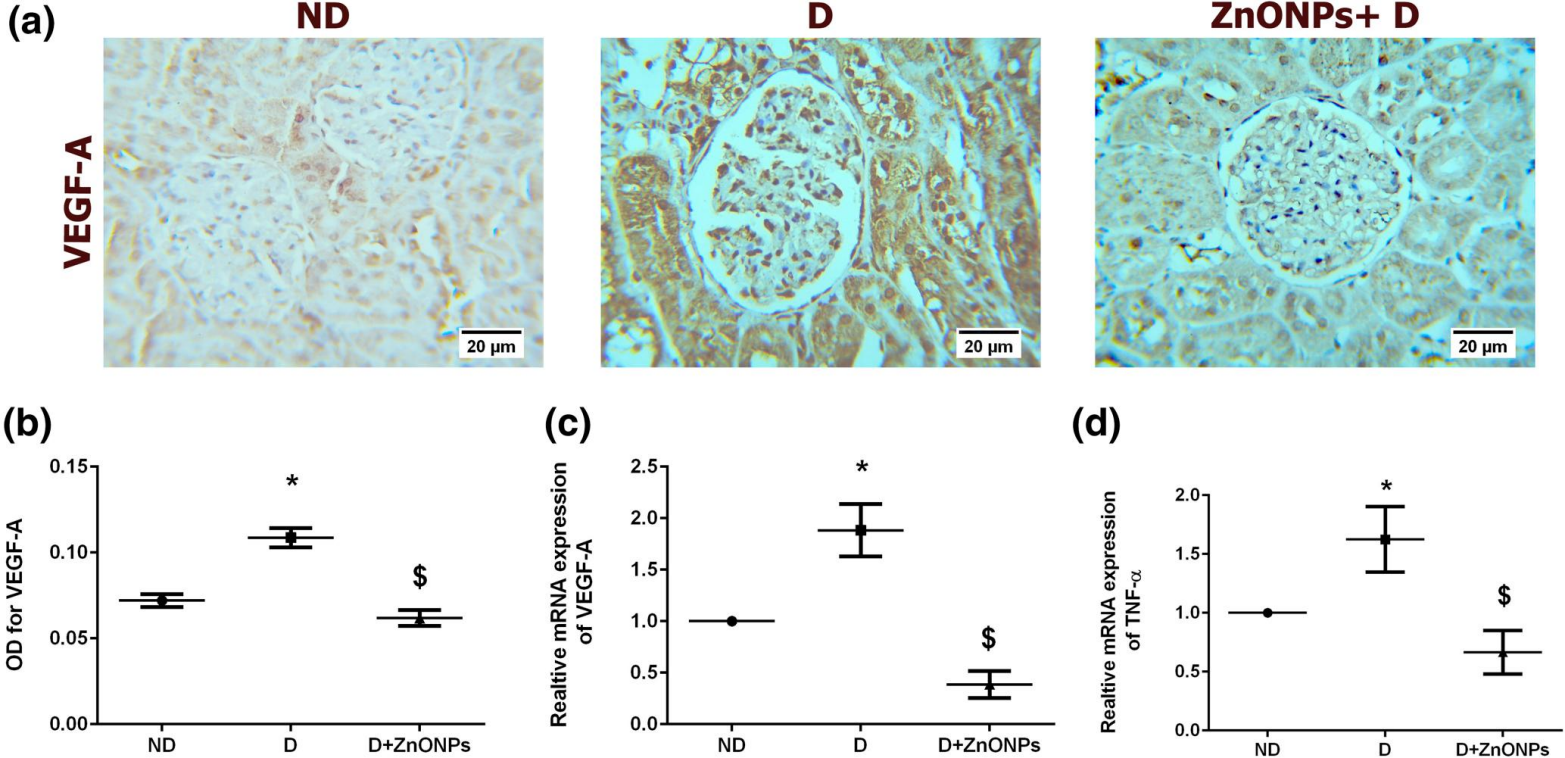
(a) Representative VEGF‐A immunohistochemical stain of the kidney sections (haematoxylin staining; magnification ×400). (b) Quantification of the immunohistochemistry results expressed as integral optical density of brown staining in the different groups. (c) mRNA expression level of VEGF‐A. (d) mRNA TNF‐*α* expression level. Data represent the mean ± SEM. Symbol (*) denotes significant differences (*P* < 0.05) with non‐diabetic, symbol ($) denotes significant differences (*P* < 0.05) with diabetic group. Abbreviations: ND, non‐diabetic; D, diabetic; ZnONPs + D, zinc oxide nanoparticle–treated diabetic group; OD, optical density; TNF‐*α*, tumour necrosis factor‐*α*; VEGEF‐A, vascular endothelial growth factor‐A

Inflammation and abnormal angiogenesis play a vital role in the pathogenesis of diabetes [6, 35]. Consistent with the effect of ZnONPs on TGF‐*β*1, the mRNA expression of TNF‐*α* was also decreased in ZnONP‐treated diabetic rats (Figure 7a, *P* < 0.05). The renal VEGF‐A protein and mRNA expression levels in the kidneys from ZnONPs‐treated rats were significantly decreased compared with the kidneys of the D group (Figure 7b–d, *P* < 0.05).

### 3.6 Zinc oxide nanoparticles inhibited the renal oxidative stress in DN rats induced by streptozotocin

**FIGURE 8:**
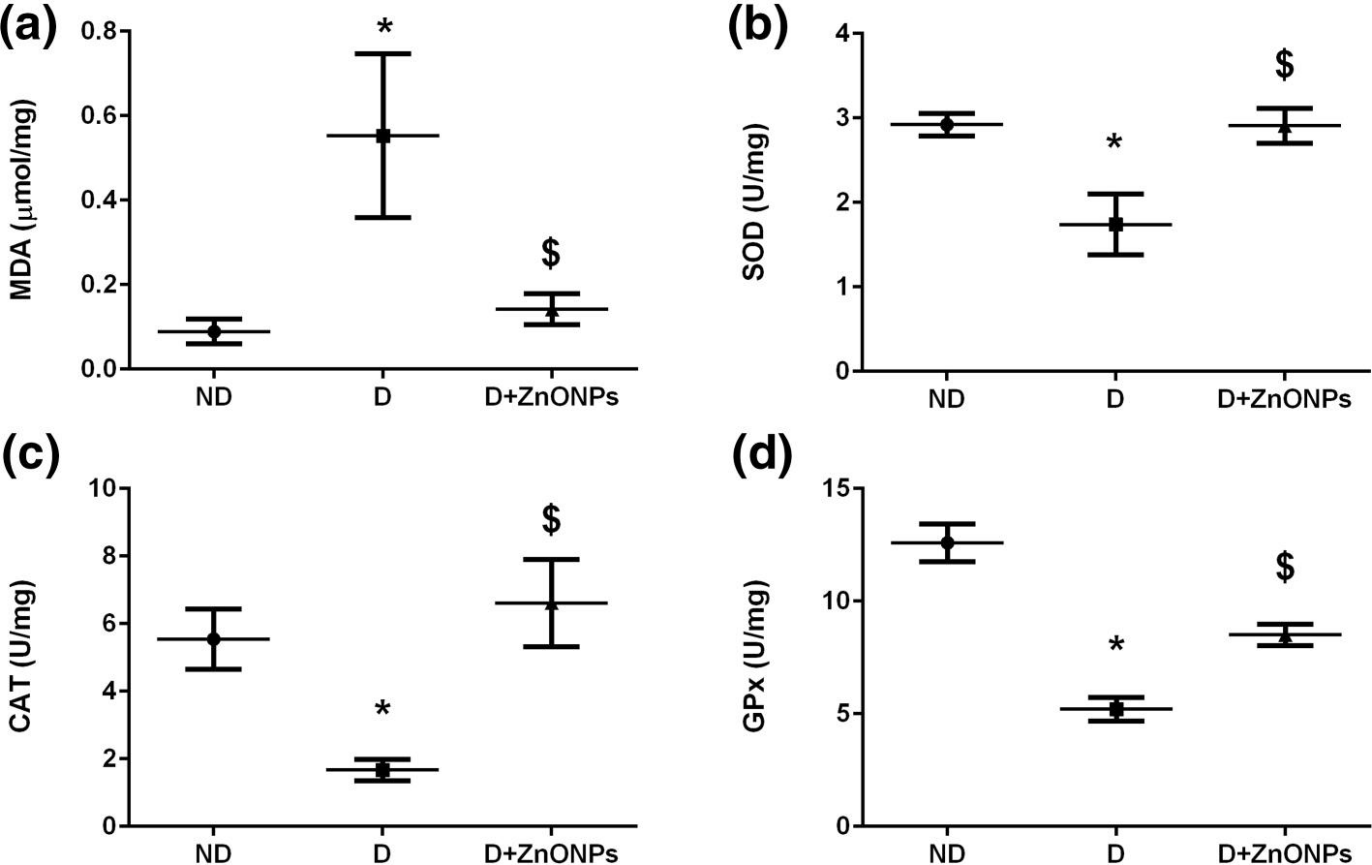
Effects of ZnONPs on different oxidative stress markers in renal tissue: (a) MDA levels (b) SOD activity levels; (c) CAT activity levels; (d) GPx activity levels. All values are expressed as mean ± SEM. Symbol (*) denotes significant differences (*P* < 0.05) with non‐diabetic, symbol ($) denotes significant differences (*P* < 0.05) with diabetic group. Abbreviations: ND, non‐diabetic; D, diabetic; ZnONPs + D, zinc oxide nanoparticle–treated diabetic group; MDA, malondialdehyde; SOD, superoxide dismutase; CAT, catalase; GPx, glutathione peroxidase

Compared with the ND rat group, renal tissue MDA level was remarkably increased (*P* < 0.05), Figure 8a, while the activities of SOD (*P* < 0.05), Figure 8b, CAT (*P* < 0.05), Figure 8c, and GPx (*P* < 0.05), Figure 8d, were significantly reduced in the diabetic rats. These effects were effectively reversed by ZnONPs treatment (*P* < 0.05).

## 4 DISCUSSION

In recent years, the use of nanomedicine has increased exponentially, being utilised for diagnosis, prevention and treatment of different diseases [15]. This study is the first to exclusively evaluate the possible therapeutic effect of ZnONPs on DN in an STZ‐induced type 1 diabetes rat model. The STZ‐induced DN rat model is a well‐characterised model of diabetes mellitus–induced renal injury characterised by albuminuria and hyperglycemia and associated with mesangial expansion, thickening of the GBM and loss of podocytes [3].

In DN, changes in GBM thickening and mesangial expansion are considered the first measurable structural changes in the glomerulus [3]. These structural changes cause a decrease in filtration surface area that leads to reduction in glomerular filtration rate [4]. On the other hand, albuminuria has been associated with podocyte injury manifested by foot process widening and effacement, decreased podocyte number (podocytopenia) and the loss of slit diaphragm proteins such as nephrin and podocin [4]. In the current study, ZnONP treatment significantly decreased GBM thickness and increased podocyte number and the expression of nephrin and podocin that resulted in decreased UAE in the diabetic rats. This suggests a potential protective effect of ZnONPs against albuminuria and podocyte injury in a rat model of DN. Consistent with our findings, previous studies have shown that three months of zinc supplementation in human patients and rats significantly reduced UAE and prevented diabetes‐induced renal pathological changes and fibrosis [36, 37].

Renal fibrosis is mainly intermediated by the pro‐fibrotic cytokine TGF‐*β*1 [38]. In addition, excessive ECM protein accumulation (collagen IV and fibronectin) results from overproduction or a decrease in ECM protein degradation by MMPs and is associated with diabetic renal fibrosis [3]. Although the exact mechanism of the anti‐fibrotic role of ZnONPs treatments in DN disease is not well understood in this study, we demonstrate that ZnONP treatment decreased the expression of TGF‐*β*1, collagen IV, and fibronectin and increased the expression of MMP‐9 in the diabetic kidney. A decline in the expression and activity of MMP‐9 contributes to mesangial matrix accumulation in the diabetic kidney [33]. Therefore, increased degradation, as well as decreased ECM protein synthesis could contribute to the observed anti‐fibrotic effects of ZnONPs treatment in DN disease.

Mitochondrial reactive oxygen species (ROS) production such as superoxide anions, hydrogen peroxide and hydroxyl radicals is found to be increased during hyperglycemia [39]. Excess ROS generation activates several signalling pathways and transcription factors, including protein kinase C, mitogen‐activated protein kinases and TGF‐*β*, which in turn causes increased expression of ECM proteins and progression to renal fibrosis [5, 39]. Thereby, restoration of the antioxidant defence system by using exogenous anti‐oxidative agents prevented ROS‐mediated diabetic kidney disease [40]. In many diseases, including DN, the antioxidant properties of Zn have been well documented [11, 13]. In the present study, ZnONPs treatment improved the renal antioxidant defence mechanisms as indicated by the decrease in MDA level and the increase in antioxidant enzymatic activities, which are responsible for protection from oxidative stress (i.e. SOD, CAT and GPx).

Abnormal angiogenesis has been identified as a significant consequence during the development of DN [6]. Therefore, therapeutic options have been proposed to prevent or delay the nephropathy process in diabetic patients by targeting angiogenic factors such as VEGF‐A and angiopoietins. The current study demonstrated an inhibitory effect of ZnONP on the angiogenesis of nephropathic diabetic rats, as shown by downregulation of the VEGF‐A marker. The anti‐angiogenic effect of ZnONP has been described in many reports [41, 42]. Our findings are consistent with these previous findings, showing the same impact of nanoparticles by preventing the formation of new blood vessels. The exact mechanism by which ZnONPs inhibit the expression of VEGF‐A is not understood. However, conformational changes in the structure of the VEGF‐A has been proposed as a mechanism of nanoparticles anti‐angiogenic effects [43].

The inflammation process is one of the significant driving mechanisms that are involved in the development of DN. TNF‐*α* is a proinflammatory cytokine that plays a pivotal role in the development of microvascular diabetic complications, including DN [44].Therapeutic strategies aiming to inhibit TNF‐*α* production showed improvement of glomerular and tubulointerstitial injury in patients with DN [44]. The anti‐inflammatory effects of ZnONPs have been described in multiple studies [17, 45, 46]. In a previous study, Min‐Ho Kim et al. reported that ZnONPs exhibited an anti‐inflammatory effect by downregulating the expression of interleukin (IL)‐1*β*, IL‐6, and TNF‐*α* [45]. Consistent with this, our findings support a potential anti‐inflammatory behaviour for ZnONPs in the prevention of DN by inhibiting the expression of inflammatory cytokines (TGF‐*β*1 and TNF‐*α*).

In conclusion, ZnONPs ameliorate the renal damage in the experimental DN rat model through the improvement of renal function, inhibition of renal fibrosis, oxidative stress, inflammation, and abnormal angiogenesis and the amelioration of podocyte injury. All data are summarised in Supplement 2. These findings may help in designing clinical applications of ZnONPs for protection against the development of DN.

## CONFLICTS OF INTEREST

The authors declare there are no conflicts of interest regarding the publication of this article.

## Figures and Tables

**Supplement 1:**
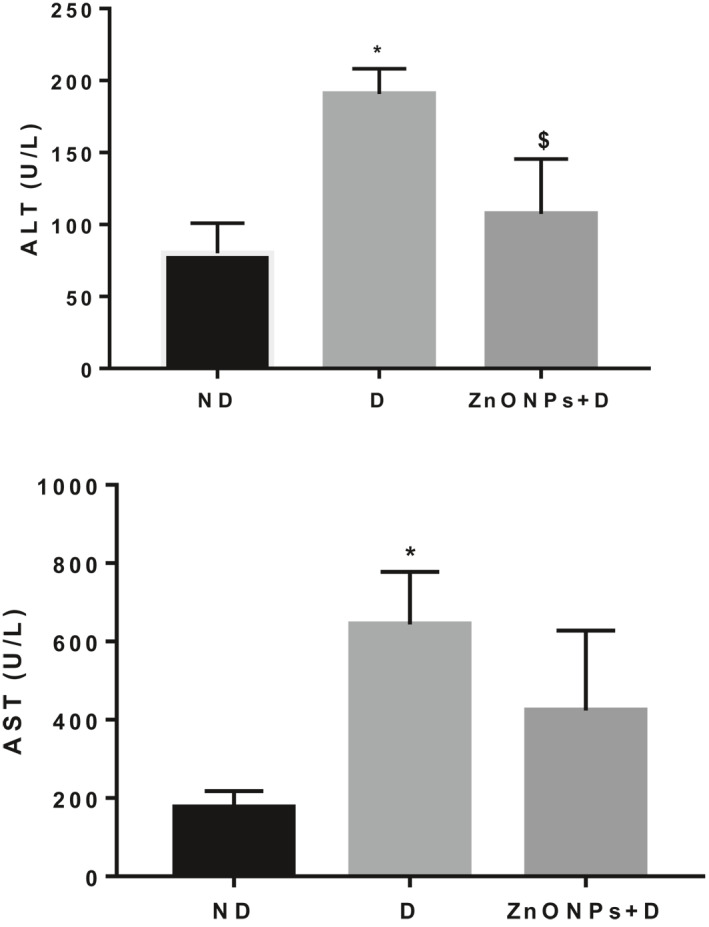
Histograms representing serum alanine aminotransferase and aspartate aminotransferase values after treating diabetic rats with zinc oxide nanoparticles

## 1

**Supplement 2:** Summary of all values in the study

Group	Non‐diabetic (ND)	Diabetic (D)	ZnONPs + D	*p* valueD versus ZnONPs + D
Parameter				
UAE rate mg/ml	0.157 ± 0.042	0.733 ± 0.058	0.2874 ± 0.059	<0.0001
Blood glucose mg/dl	113.7 ± 4.784	370.6 ± 23.7	194.7 ± 49.64	0.0012
BUN levels mg/dl	44.77 ± 1.12	128.4 ± 5.694	77.04 ± 18.76	0.0059
KWI	0.6336 ± 0.019	1.161 ± 0.037	0.8006 ± 0.134	0.0067
Renal tissue SOD activity (U/mg)	2.921 ± 0.1337	1.739 ± 0.360	2.907 ± 0.205	0.0095
Renal tissue GPx activity (U/mg)	12.59 ± 0.836	5.208 ± 0.526	8.5 ± 0.483	0.0021
Renal tissue CAT activity (U/mg)	5.544 ± 0.894	1.67 ± 0.314	6.611 ± 1.295	0.0019
Renal tissue malondialdehyde (MDA) level (µmol/mg)	0.0893 ± 0.029	0.553 ± 0.194	0.1417 ± 0.037	0.0232
relative TGF‐*β*1 mRNA expression level	1 ± 0	1.642 ± 0.221	0.697 ± 0.092	<0.0001
Relative VEGF‐A mRNA expression level	1 ± 0	1.883 ± 0.2546	0.3864 ± 0.1319	<0.0001
Relative MMP‐9 mRNA expression level	1 ± 0	0.275 ± 0.06	0.8205 ± 0.2396	0.0251
Relative fibronectin mRNA expression level	1 ± 0	1.858 ± 0.439	0.07833 ± 0.044	0.0003
Relative TNF‐*α* mRNA expression level	1 ± 0	1.624 ± 0.278	0.6654 ± 0.185	0.0025

Note: Each value is expressed as mean ± SEM.

Abbreviations: BUN, blood urea nitrogen; CAT, catalase; GP, glutathione peroxidase; KWI, Kidney weight index; SOD, superoxide dismutase; UAE, Urinary albumin excretion.
